# Comparison of the effects of cognitive-supportive-relaxation multimodal psychosocial intervention on fear of cancer recurrence and psychological resilience in breast cancer patients: a network meta-analysis based on randomized controlled trials

**DOI:** 10.3389/fonc.2026.1597317

**Published:** 2026-02-27

**Authors:** Yuan Jiankun, Li Xiaojuan, Wang Liyi, Dai Xuejun, Deng Aihua, He Lang

**Affiliations:** Department of Oncology, Chengdu Fifth People’s Hospital (The Fifth People's Hospital Affiliated to Chengdu University of Traditional Chinese Medicine), Chengdu, Sichuan, China

**Keywords:** breast cancer, cognitive behavioral therapy, fear of cancer recurrence, network meta-analysis, psychological resilience

## Abstract

**Background:**

Fear of cancer recurrence (FCR) is a highly prevalent and clinically significant psychological concern among breast cancer patients, which can substantially impair their physical and psychological well-being. Emerging evidence suggests that psychotherapeutic interventions exert heterogeneous effects on FCR and psychological resilience in this patient population; however, a paucity of comprehensive network meta-analyses has systematically compared the relative efficacy of diverse psychotherapies for FCR in breast cancer survivors. The present network meta-analysis was conducted to systematically evaluate and compare the effectiveness of various mind-body interventions in mitigating FCR and enhancing psychological resilience among breast cancer patients.

**Methods:**

Randomized controlled trials (RCTs) on different psychotherapeutic methods for psychological problems such as the fear of cancer recurrence, anxiety, and depression in breast cancer patients were retrieved by computer from PubMed, Embase, Cochrane Library, Web of Science, CNKI (China National Knowledge Infrastructure), VIP Database for Chinese Technical Periodicals, Wanfang Data Knowledge Service Platform, and Chinese Biomedical Literature Database. To ensure the novelty of the research, the retrieval time limit was set from January 2019 to March 2025. Two researchers screened the literature, extracted the data, and evaluated the methodological quality according to the inclusion and exclusion criteria. Network meta-analysis was conducted using RevMan 5.4 and Stata 17.0.

**Results:**

Nineteen eligible randomized controlled trials (RCTs) involving 11 interventions and 2214 participants were included. Network meta-analysis indicated distinct psychotherapeutic approaches had superior efficacy for specific psychological outcomes: couple skills training showed probabilistic advantages for fear of cancer recurrence (FCR, Fear of Cancer Recurrence Inventory); narrative therapy was effective for recurrence-related concerns; cognitive behavioral therapy (CBT) had probabilistic benefits in reducing fear of progression (Fear of Progression Questionnaire-Short Form) and enhancing positive psychological capital (Positive Psychological Capital Questionnaire); and both CBT and mindfulness therapy outperformed other interventions for anxiety and depression (Hospital Anxiety and Depression Scale). Sensitivity and subgroup analyses further validated the robustness of the findings and explored sources of heterogeneity. Subgroup analysis identified measurement time points and treatment status as the primary contributors to substantial overall heterogeneity (I²=91.9%, P<0.001), whereas measurement tools had no notable effect;leave-one-out sensitivity analysis showed the pooled effect size stably ranged from -1.44 to -0.63, with no single study driving overall results, confirming the robustness of core conclusions.

**Conclusion:**

Our findings underscore the outcome-specific advantages of different psychotherapeutic interventions for breast cancer patients, thereby supporting the adoption of a personalized, needs-based approach to address FCR, psychological distress, and resilience. Nevertheless, the current evidence is constrained by substantial heterogeneity in intervention protocols, variability in outcome measurement tools, and a relatively limited number of trials for certain interventions. Future high-quality RCTs with standardized intervention protocols, consistent outcome metrics, and long-term follow-up are warranted to validate these findings and inform the development of clinical practice guidelines for psychological support in breast cancer survivors.

**Systematic review registration:**

https://www.crd.york.ac.uk/PROSPERO/, identifier CRD420251009772.

## Background

Breast cancer (BC), as one of the most common malignant tumors among women worldwide, seriously threatens women’s physical and mental health. According to the data on the global cancer burden in 2020 released by the International Agency for Research on Cancer (IARC) of the World Health Organization, the number of new breast cancer cases reached as high as 2.26 million, exceeding the 2.2 million new cases of lung cancer and ranking first among the newly diagnosed cancer cases globally (1). With the continuous progress of medical technology, the survival rate of breast cancer patients has significantly increased. However, the psychological problems after treatment have gradually become prominent and have become an important factor affecting the quality of life and the rehabilitation process of patients.

The fear of cancer recurrence (FCR) is a prevalent and prominent psychological issue among breast cancer patients. The latest research shows that approximately 40%-70% of breast cancer patients will be troubled by FCR after treatment (2). FCR not only exacerbates psychological problems such as anxiety and depression but also, to a certain extent, reduces the treatment compliance of patients (3). At the same time, psychological resilience, as an individual’s positive adaptive ability to cope with trauma, is considered a key protective factor for alleviating FCR (4). Therefore, enhancing the psychological resilience of breast cancer patients can help them reduce anxiety and depression, thereby alleviating the fear of cancer recurrence and achieving the effect of improving the long-term prognosis.

Different psychotherapeutic methods have varying impacts on the fear of cancer recurrence and psychological resilience in BC patients. Currently, there is a lack of research on the influence of different psychotherapies on the fear of cancer recurrence in BC patients. Therefore, this study conducts a systematic review of the effects of different psychotherapies on the fear of cancer recurrence and psychological resilience in BC patients, which can provide certain references and insights for the optimal selection of psychotherapies for the fear of cancer recurrence and psychological resilience in BC patients.

## Methods

### Search strategy

The method of combining subject headings and free words was adopted to search for randomized controlled trials (RCTs) published from January 2019 to March 2025 in the Cochrane Library, PubMed, Embase, Web of Science, CNKI (China National Knowledge Infrastructure), Wanfang Data Knowledge Service Platform, VIP Chinese Science and Technology Journal Full-text Database, and Chinese Biomedical Literature Database. A review protocol was registered with the International Prospective Register of Systematic Reviews (PROSPERO), registration number CRD420251009772. These RCTs focused on the therapeutic effects of different psychotherapies on the fear of cancer recurrence and psychological resilience in breast cancer patients. English search terms included “breast cancer/mammography”, “fear of cancer recurrence/fear of recurrence/fear of disease progression”, “cognitive behavioral therapy/mindfulness stress reduction/relaxation training/social support/supportive intervention”, “psychological elasticity/mental toughness/resilience”.

Taking PubMed as an example, the specific retrieval strategy was: (((“Breast Neoplasms”[Mesh]) OR breast cancer[tiab])) AND ((“Fear of Cancer Recurrence”[Mesh]) OR “fear of cancer recurrence”[tiab]) AND ((“Psychological Resilience”[Mesh]) OR “psychological resilience”[tiab]) AND (“Randomized Controlled Trials as Topic”[Mesh]) AND ((“Cognitive Therapy”[Mesh]) OR “cognitive therapy”[tiab]) AND ((“Supportive Psychotherapy”[Mesh]) OR “supportive psychotherapy”[tiab]) AND ((“Relaxation Training”[Mesh]) OR “relaxation training”[tiab]) AND ((“Multimodal Treatment”[Mesh]) OR “multimodal treatment”[tiab]) AND ((“Meta-Analysis as Topic”[Mesh]) OR “meta-analysis “[tiab]).

### Inclusion criteria

The criteria for inclusion in the eligible articles were as follows:(1) Research subjects: Breast cancer patients; (2) Research type: Randomized controlled trial (RCT); (3) Intervention measures: In the study group, psychotherapy different from that of the control group is adopted; In the control group, routine psychotherapy or psychotherapy different from that of the study group is adopted; (4) Outcome indicators: Fear of cancer recurrence (FCR), psychological resilience, anxiety, and depression.

### Exclusion criteria

Exclusion criteria included: (1) Literature whose research types do not match; (2) Literature for which the full text cannot be obtained or that has been repeatedly published; (3) Literature in which the data are reused or incorrect; (4) Literature from which the data cannot be extracted; (5) Literature that is not within the specified retrieval time limit.

### Literature screening

Two researchers screened the literature according to the inclusion and exclusion criteria. The Zotero software was used to remove duplicate literature. The remaining literature was initially screened based on their titles and abstracts, and then a full-text reading was conducted for re-screening. DXJ and DAH independently undertook literature screening and data extraction. Subsequently, they carefully perused the full texts and re-screened them in strict accordance with the pre-established inclusion and exclusion criteria. After that, the two researchers convened to deliberate on the screening outcomes. When they reached a consensus, the corresponding study was incorporated. In cases where there was a lack of agreement, a third researcher was invited to participate. Through in-depth discussion and the attainment of a mutual understanding, a collective final decision was made.

### Data extraction

DXJ and DAH independently extracted data such as the first author, publication time, country, sample size, cancer stage/treatment status, measurement time points, intervention methods, and outcome indicators. The data were then double - checked by the two researchers.

### Risk of bias assessment

Two researchers independently assessed the risk of bias in the included RCTs using the Cochrane Handbook for Systematic Reviews of Interventions (Version 5.1.0) (5). Disputes were resolved through consultation with a third researcher, who was consulted for discussion and decision - making. This assessment standard consists of 7 items, and the researchers were required to make judgments of “low risk of bias”, “high risk of bias”, or “unclear” for each item.

### Statistical analysis

RevMan 5.4 was used for literature quality assessment, and Stata 17.0 was employed for network meta-analysis and graphing. When the evidence network forms a closed loop, Stata 17.0 is used to conduct a global consistency test; and the node-splitting method was adopted for the inconsistency test. If P>0.05, it indicated no significant inconsistency and a consistency model was used for the analysis (6). For continuous variables measured by the same instrument, the weighted mean difference (WMD) was calculated. For those measured by different instruments, the standardized mean difference (SMD) was calculated. Additionally, a 95% confidence interval (CI) was computed for each effect size. This study adhered to the Preferred Reporting Items for Systematic Reviews and Meta-Analyses for Network Meta-Analysis (PRISMA- NMA).Then, a surface under the cumulative ranking (SUCRA) plot was drawn. The larger the SUCRA value, the better the intervention effect. A bias funnel plot was used to observe publication bias.

### Transitivity and clinical comparability assessment

Transitivity represents a foundational assumption underpinning the validity of network meta-analysis, which mandates that the distribution of key effect modifiers—including patient characteristics, intervention-related features, and outcome measurement approaches—is balanced across all pairwise comparisons within the network (46). In the present study, we formally evaluated the transitivity assumption by examining treatment status (a patient characteristic), post-intervention measurement time points (an intervention characteristic), and the measurement tools employed for outcome assessment. Concomitantly, to mitigate potential transitivity violations and account for heterogeneity arising from these modifiers, we conducted prespecified subgroup analyses stratified by measurement tools, measurement time points, and treatment status, where analytically feasible.

## Results

### Literature screening process and results

**Figure 1:**
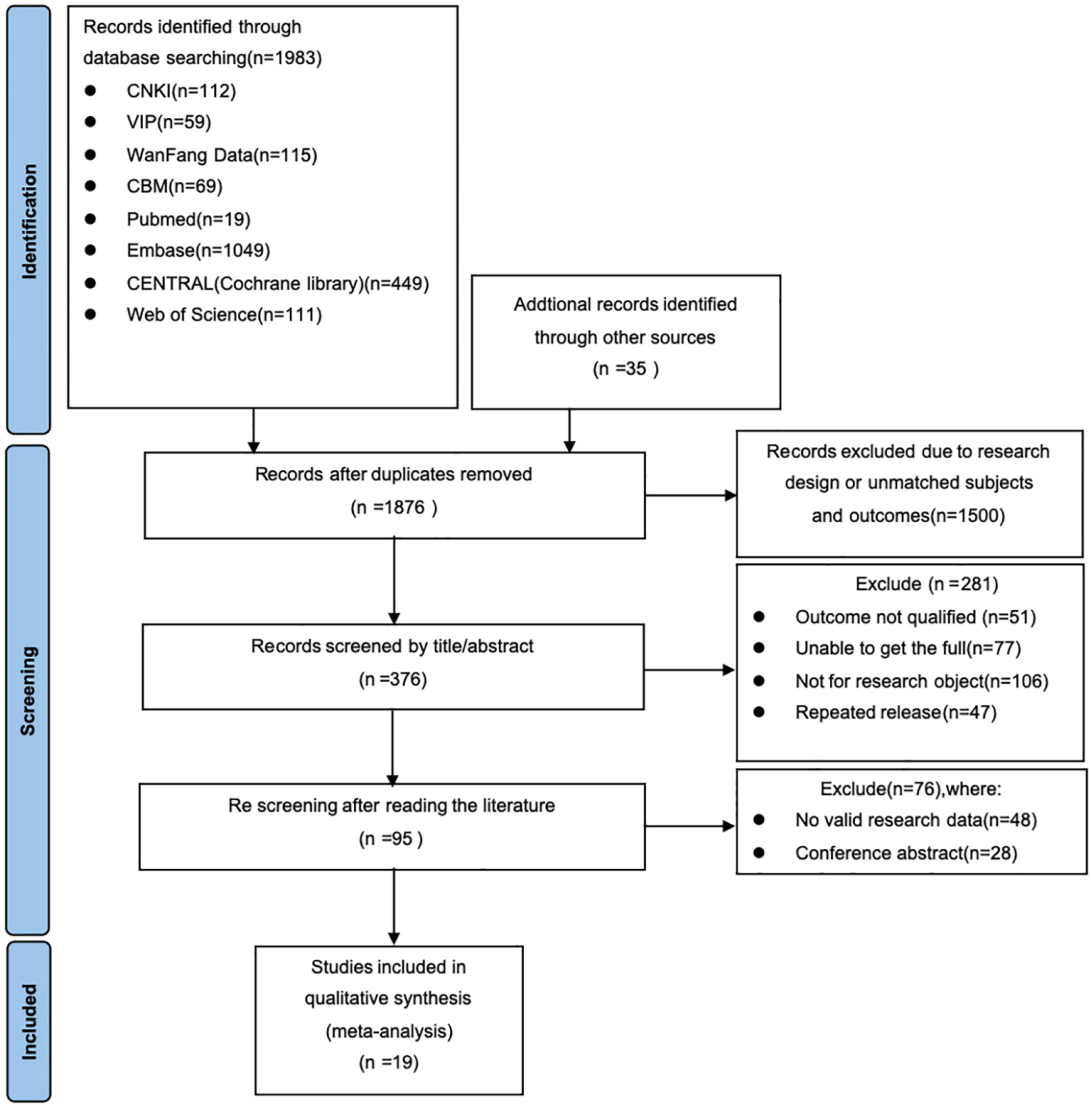
Flow chart of literature screening.

A total of 2,018 literatures were retrieved, including 355 in Chinese and 1,628 in English. Additionally, 35 kinds of literature were obtained through other retrieval channels. After a thorough review of the full texts, 1,999 literatures were excluded due to various reasons. Consequently, 19 kinds of literature were finally included, among which 11 were in Chinese and 8 were in English. The total sample size was 2,214 cases, involving 11 different types of psychotherapies. The literature screening process is depicted in **Figure 1**.

### Basic characteristics of the included studies

The study group received different types of psychotherapies. Specifically, 4 studies involved PERMA Positive Psychotherapy (7, 9, 13, 14), 5 studies involved Mindfulness therapy (12, 15, 16, 24, 25), 1 study involved Self - disclosure in Positive Psychology (8), 1 study involved Narrative Therapy (10), 2 studies involved Cognitive - behavioral therapy (11, 22), 1 study involved Digital Psychotherapy (23), 2 studies involved Couples’ skills training (17, 18), 2 studies involved Group Psychotherapy (19, 21), 1 study involved Relaxation training (19), and 1 study involved Comprehensive Psychosocial Therapy: Nurse Navigation Intervention (20).

In addition, 8 studies used the FCRI scale, 7 studies used the FoP - Q - SF scale, 3 studies used the PPQ scale, 3 studies used the CARS scale, and 5 studies used the HADS scale. These studies were conducted across different countries and regions. Among them, 13 studies were from China, 2 from Denmark, 2 from Japan, 1 from Belgium, and 1 from the United Kingdom. **Table 1** delineates the fundamental attributes of the incorporated literature.

**Table 1:** Characteristics of the studies included in the NMA.

Study ID	Year	Country	Design	(Cancer stage/ treatment status)	Intervention (T vsC)	Number of cases	Outcome indicators	Measurement time points
Wang Juan (7)	2024	CHN	RCT	BC 0-III/a+b+c	A/C	110	①②	Post-intervention 8 weeks
Chen Jiao (8)	2022	CHN	RCT	BC 0-III/b	B/C	88	③	Post-intervention 8 weeks
Dong Cuili (9)	2022	CHN	RCT	BC 0-III/a+b	A/C	114	③④	Post-intervention 8 weeks
Gao Xueyun (10)	2022	CHN	RCT	BC 0-III/b	D/C	90	⑤	Post-intervention 8 weeks
Deng Juan (11)	2022	CHN	RCT	BC 0-III/a	E/C	90	③④	Post-intervention 8 weeks
Wang Xiaomei (12)	2021	CHN	RCT	BC 0-III/a+b+c	G/C	70	①⑥	Post-intervention 8 weeks
Dong Shuxian (13)	2021	CHN	RCT	BC 0-III/b	A/C	87	③	Post-intervention 12 weeks
Wang Jiajia (14)	2021	CHN	RCT	BC 0-III/a	A/C	60	③④	Post-intervention 8 weeks
Wang Jinmei (15)	2021	CHN	RCT	BC 0-III/a	G/C	88	⑤	Post-intervention 6 weeks
Zhou Jiaojiao (16)	2020	CHN	RCT	BC 0-III/a	G/C	99	③	Post-intervention 8 weeks
Zhang Xianxian (17)	2019	CHN	RCT	BC 0-III/b	H/C	61	③	Post-intervention 12 weeks
Cheng, Y (18)	2023	CHN	RCT	BC 0-III/b	H/C	90	①	Post-intervention 13 weeks
Tauber, NM (19)	2023	DK	RCT	BC 0-III/a+b+c	I/J	85	①	Post-intervention 1 weeks
Bidstrup, PE (20)	2023	DK	RCT	BC 0-III/a+b+c	K/C	309	①	Post-intervention 26 weeks
Merckaert, I (21)	2023	BE	RCT	BC 0-III/a	I/C	120	①⑥	Post-intervention 21 weeks
Akkol-Solakoglu (22)	2023	UK	RCT	BC 0-III/a+b+c	E/C	72	⑥⑦	Post-intervention 8 weeks
Akechi, T (23)	2023	JP	RCT	BC 0-III/a	F/C	447	⑥①	Post-intervention 8 weeks
Peng L (24)	2022	CHN	RCT	BC 0-III/b+c	G/C	60	①	Post-intervention 6 weeks
Park, S (25)	2020	JP	RCT	BC 0-III/a+b+c	G/C	74	⑤⑥	Post-intervention 8 weeks

A, PERMA Positive Psychotherapy(PERMA); B, Self-disclosure in Positive Psychology(SDPP); C, Treatment as usual(TAU); D, Narrative Therapy(NT); E, Cognitive-behavioral therapy(CBT); F, Digital Psychotherapy(DP); G, Mindfulness therapy(MT); H, Couples’s skills training(CST); I, Group Psychotherapy(GP); J, Relaxation training(RT); K, Comprehensive Psychosocial Therapy; Nurse Navigation Intervention(CPT, NNI).

a, Surgical treatment; b, Chemotherapy; c, Radiation therapy.

①FCRI, The Fear of CancerRecurrence Inventory; ②BCRS, Breast Cancer Patients’ Psychological Resilience Scale; ③Fop-Q-SF, Fear of Progression Questionnaire-Short Form; ④PPQ, Positive Psychological Capital Questionnaire; ⑤CARS, The Concerns About Recurrence Scale; ⑥HADS, Hospital Anxiety and Depression Scale; ⑦CWS, The Cancer Worry Scale.

BC 0-III, Stage 0–III breast cancer.

### Quality assessment of the included studies

**Figure 2:**
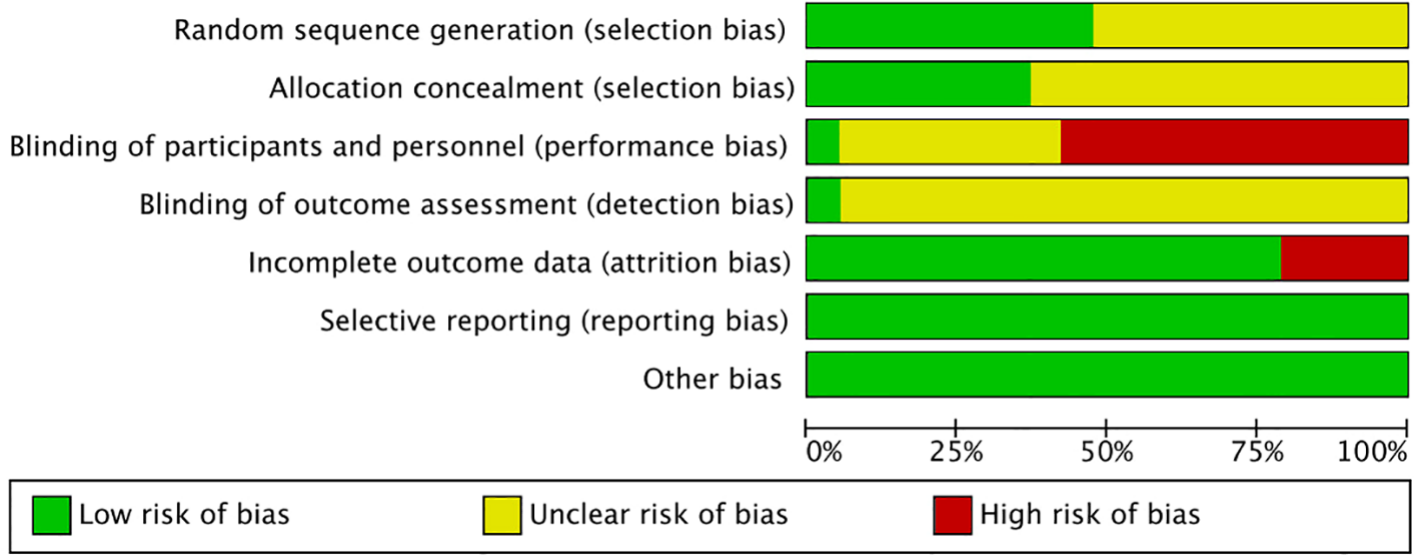
Risk of bias graph.

Among the 19 studies, 9 reported generating random sequences via random number tables or computer-based approaches, while 7 described allocation concealment methods. However, only 1 study specified a blinding strategy, resulting in notable performance and detection bias. Regarding data integrity, 15 studies demonstrated good practices, and no selective reporting bias was identified across all included trials. Collectively, these assessments indicate that most RCTs were at moderate risk of bias, with full details of the risk-of-bias evaluation presented in **Figure 2**.

### Network meta-analysis

#### Evidence network and coherence analysis

**Figure 3:**
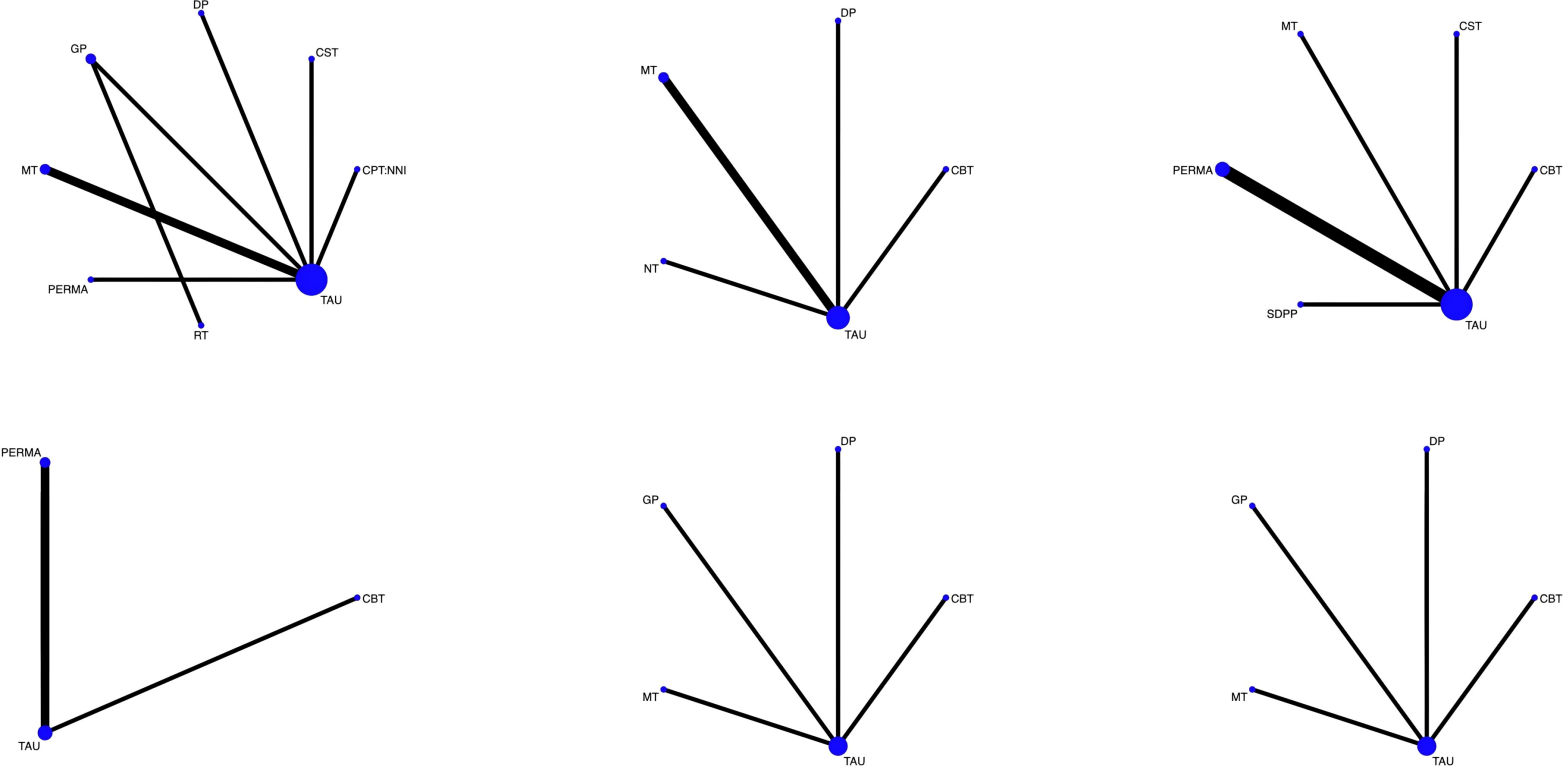
Network graph of interventions.

The NMA results are shown in **Figure 3**. Eight studies (7, 12, 18–21, 23, 24) investigated the impacts of various psychotherapies on the fear of cancer recurrence among BC patients. The six psychotherapeutic approaches covered in these studies were PERMA-positive psychotherapy, mindfulness-based therapy, couple skills training, group psychotherapy, digital-based psychotherapy, and comprehensive psychosocial intervention in the form of nurse navigation. A total of seven direct comparisons were made. The statistical analysis revealed that P = 0.1913>0.05, suggesting that the inconsistency model did not reach statistical significance. Consequently, we adopted the consistency model for further study. In all local inconsistency tests, the P - values were greater than 0.05, indicating the absence of local inconsistency. Since no circular structure emerged among the intervention methods in this outcome, the circular inconsistency test was not conducted.

Five papers (10, 15, 22, 23, 25) explored the effects of different psychotherapies on BC patients’ worry about cancer recurrence. Four types of psychotherapies were involved, namely narrative therapy, mindfulness-based therapy, cognitive-behavioral therapy, and digital-based psychotherapy. Four sets of direct comparisons were established. The results showed that P = 0.0799 > 0.05, demonstrating that the inconsistency model was not statistically significant. Thus, we employed the consistency model for our analysis. All P - values in the local inconsistency tests were > 0.05, signifying no local inconsistency. Given that there was no circular pattern among the intervention methods in this result, the circular inconsistency test was omitted.

Seven articles (8, 9, 11, 13, 14, 16, 17) examined the influence of diverse psychotherapies on the progression of fear of cancer recurrence in BC patients. Five types of psychotherapies were included, such as positive psychological self-disclosure, PERMA positive psychotherapy, cognitive-behavioral therapy, mindfulness-based therapy, and couple skills training. Five direct comparison categories were formed. The inconsistency test yielded a result of P = 0.000<0.05, indicating a significant inconsistency. Therefore, the inconsistency model was utilized. In all local inconsistency tests, the P - values were > 0.05, suggesting no local inconsistency.

Three reports (9, 11, 14) analyzed the effects of different psychotherapies on the positive psychological capital scores of BC patients. Two psychotherapeutic modalities, PERMA-positive psychotherapy, and cognitive-behavioral therapy, were considered. Two types of direct comparisons were constructed. The inconsistency test result was P = 0.000<0.05, indicating a significant inconsistency. As a result, the inconsistency model was applied. All P - values in the local inconsistency tests were > 0.05, showing no local inconsistency.

Four pieces of research (21–23, 25) studied the impacts of various psychotherapies on the anxiety and depression scores of BC patients. Four types of psychotherapies, namely group psychotherapy, cognitive-behavioral therapy, digital-based psychotherapy, and mindfulness-based therapy, were involved. Four sets of direct comparisons were made. The inconsistency test results were P = 0.0046 < 0.05 and P = 0.0001 < 0.05, indicating significant inconsistency. Hence, the inconsistency model was used. All P - values in the local inconsistency tests were > 0.05, indicating no local inconsistency.

From left to right, the first row consists of the FCRI network evidence map, the cancer recurrence concern network evidence map, and the Fop-Q-SF network evidence map respectively. In the second row, from left to right, are the network evidence map of the PPQ (Positive Psychological Capital Questionnaire), the network evidence map of the HADS (Hospital Anxiety and Depression Scale)-Anxiety, and the network evidence map of the HADS-Depression respectively.

### Results of the network meta-analysis

#### Fear of cancer recurrence (FCRI)

**Figure 4:**
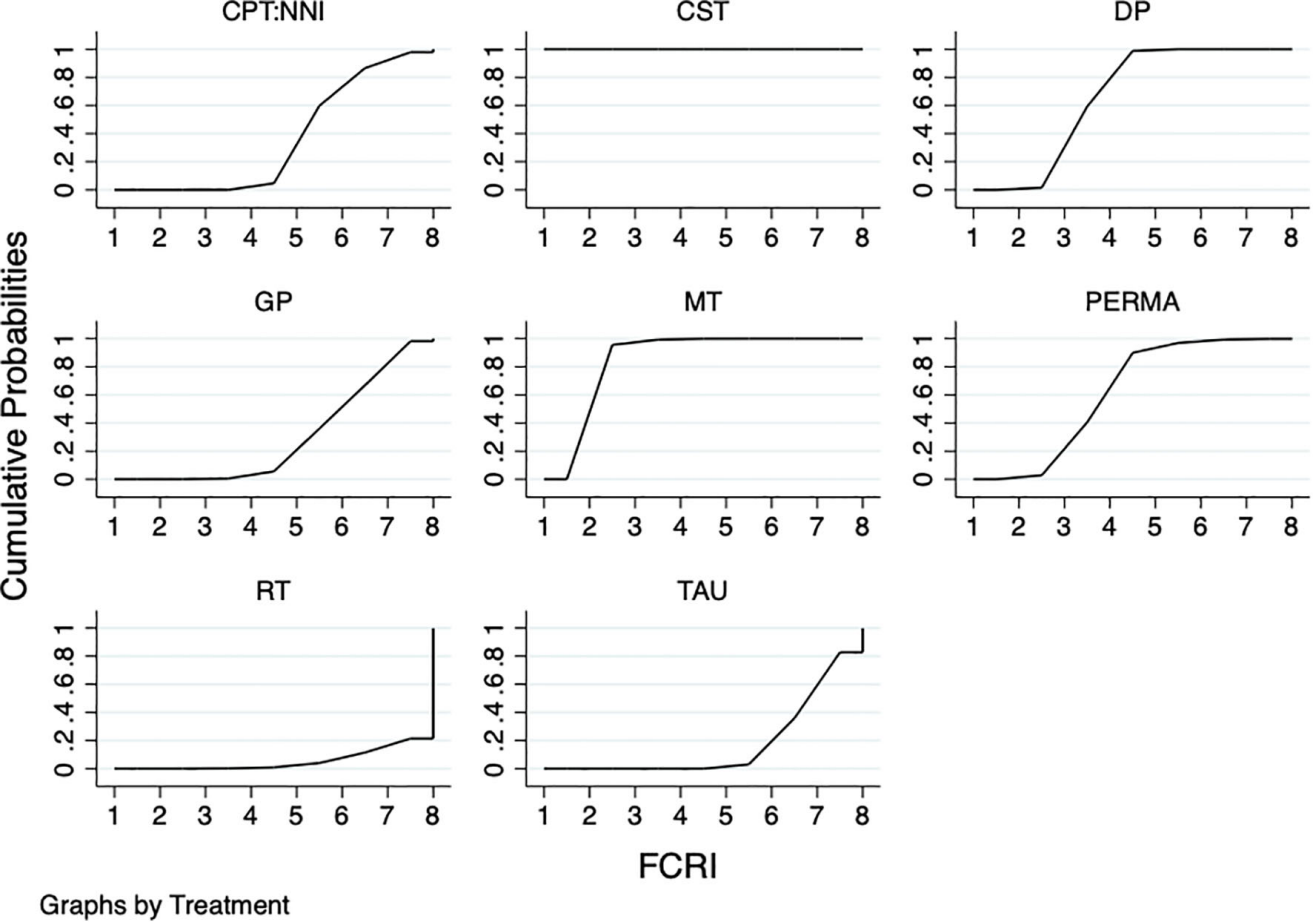
SUCRA plot of the improving fear of cancer recurrence by different psychotherapies. The SUCRA score represents only a probability ranking and does not indicate clinical superiority.

The results of the network meta-analysis showed that: compared with TAU, PERMA(SMD=-0.50,95%CI:-0.88~-0.12),CST(SMD=-3.11,95%CI:-3.73~-2.49),DP(SMD=-0.55, 95%CI: -0.74~-0.36), and MT(SMD=- 0.98,95%CI:-1.35~-0.61) were all superior to TAU, and the differences were statistically significant (P<0.05). No statistically significant differences were observed between the remaining psychotherapies and TAU (P>0.05). The results of the SUCRA probability ranking showed that CST(100%) > MT(85.0%) > DP(65.7%) > PERMA (61.4%) >CPT: NNI(35.6%)>GP(29.6%) > TAU(17.4%) > RT(5.4%) (**Table 2**; **Figure 4**).

**Table 2:** NMA of different psychotherapies on fear of cancer recurrence.

Intervention	PERMA	GP	CST	RT	DP	MT	CPT:NNI	TAU
PERMA	—	-0.41 (-0.93, 0.11)	2.61 (1.88, 3.34)	-0.75 (-1.42, -0.07)	0.05 (-0.37, 0.47)	0.48 (-0.05, 1.01)	-0.35 (-0.79, 0.09)	-0.50 (-0.88, -0.12)
GP	—	—	3.02 (2.30, 3.74)	-0.34 (-0.76, 0.09)	0.46 (0.05, 0.86)	0.89 (0.37, 1.40)	0.06 (-0.36, 0.48)	-0.09 (-0.45, 0.27)
CST	—	—	—	-3.36 (-4.19, -2.52)	-2.56 (-3.21, -1.91)	-2.13 (-2.86, -1.41)	-2.96 (-3.62, -2.30)	-3.11 (-3.73, -2.49)
RT	—	—	—	—	0.80 (0.21, 1.38)	1.22 (0.55, 1.89)	0.40 (-0.20,1.00)	0.25 (-0.31, 0.81)
DP	—	—	—	—	—	0.43 (0.01, 0.84)	-0.40 (-0.69, -0.10)	-0.55 (-0.74, -0.36)
MT	—	—	—	—	—	—	-0.83 (-1.26, -0.39)	-0.98 (-1.35, -0.61)
CPT:NNI	—	—	—	—	—	—	—	-0.15 (-0.37, 0.07)

PERMA, PERMA Positive Psychotherapy; GP, Group Psychotherapy; CST, Couples’s skills training; RT, Relaxation training; DP, Digital Psychotherapy; MT, Mindfulness therapy; CPT: NNI, Comprehensive Psychosocial Therapy: Nurse Navigation Intervention; TAU, Treatment as usual.

#### Concern about cancer recurrence(CWS/CARS)

**Figure 5:**
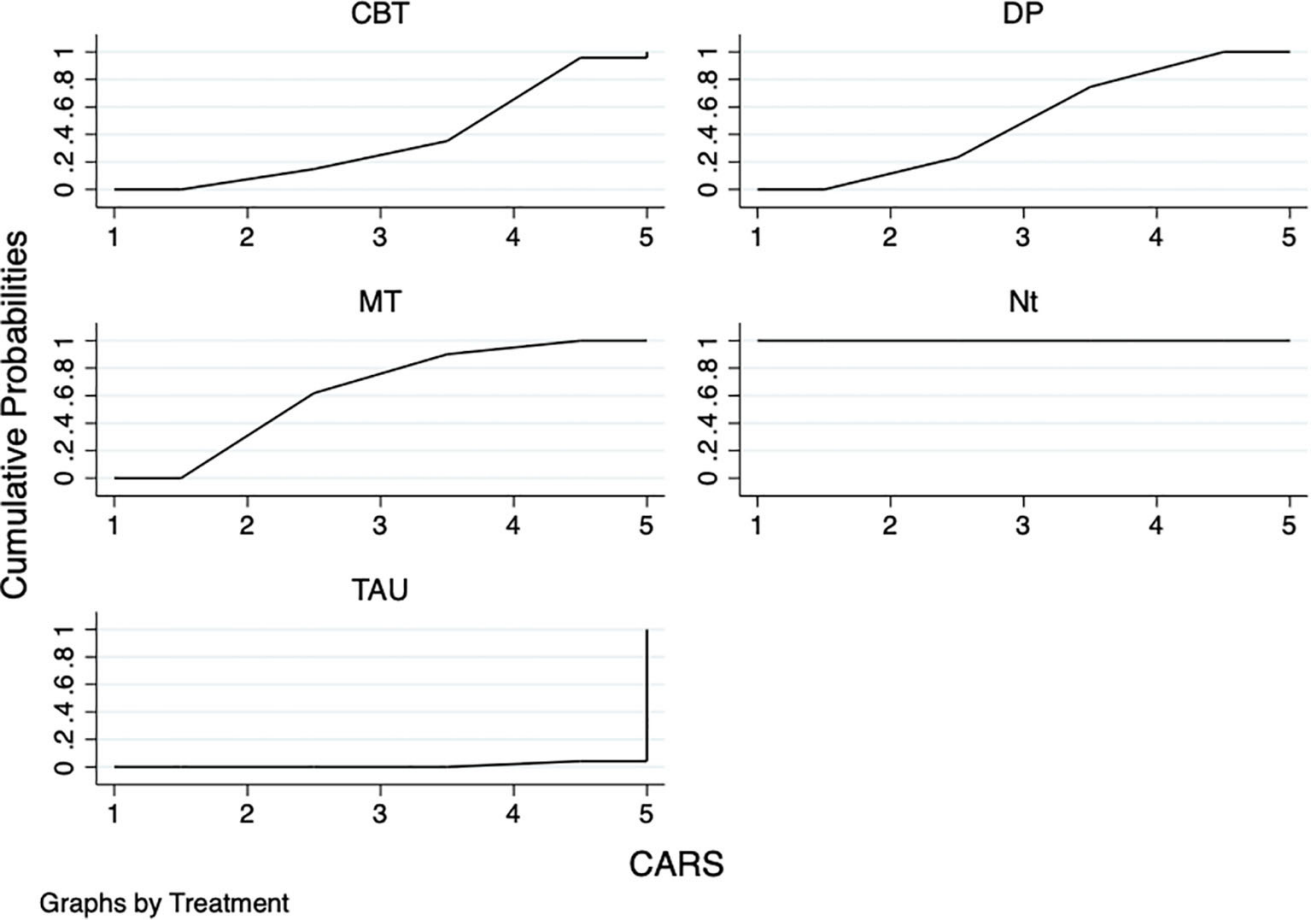
SUCRA plot of different psychotherapies for improving concerns about cancer recurrence. The SUCRA score represents only a probability ranking and does not indicate clinical superiority.

The results of the network meta-analysis showed that: compared with TAU, NT (SMD=-3.51,95%CI:-4.20~-2.81), DP(SMD=-0.64,95%CI:-0.90~-0.38), and MT (SMD=-0.77,95%CI:-1.11~-0.42) were all superior to TAU, and the differences were statistically significant (P<0.05). In comparing the remaining psychotherapies with TAU, there were no statistically significant differences (P>0.05). The results of the SUCRA probability ranking showed that NT (100%) > MT(63.0%) > DP (49.5%) > CBT(36.5%) > TAU (1.1%) (**Table 3**; **Figure 5**).

**Table 3:** NMA of different psychotherapies on cancer recurrence concerns.

Intervention	NT	DP	MT	CBT	TAU
NT	—	-2.87 (-3.61, -2.13)	-2.74 (-3.51, -1.97)	-3.03 (-3.90, -2.16)	-3.51 (-4.20, -2.81)
DP	—	—	0.13 (-0.31, 0.56)	-0.16 (-0.76, 0.43)	-0.64 (-0.90, -0.38)
MT	—	—	—	-0.29 (-0.93, 0.35)	-0.77 (-1.11, -0.42)
CBT	—	—	—	—	-0.48 (-1.01, 0.06)

NT, Narrative Therapy; DP, Digital Psychotherapy; MT, Mindfulness therapy; CBT, Cognitive-behavioral therapy; TAU, Treatment as usual.

#### Fear of progression questionnaire-short form

**Figure 6:**
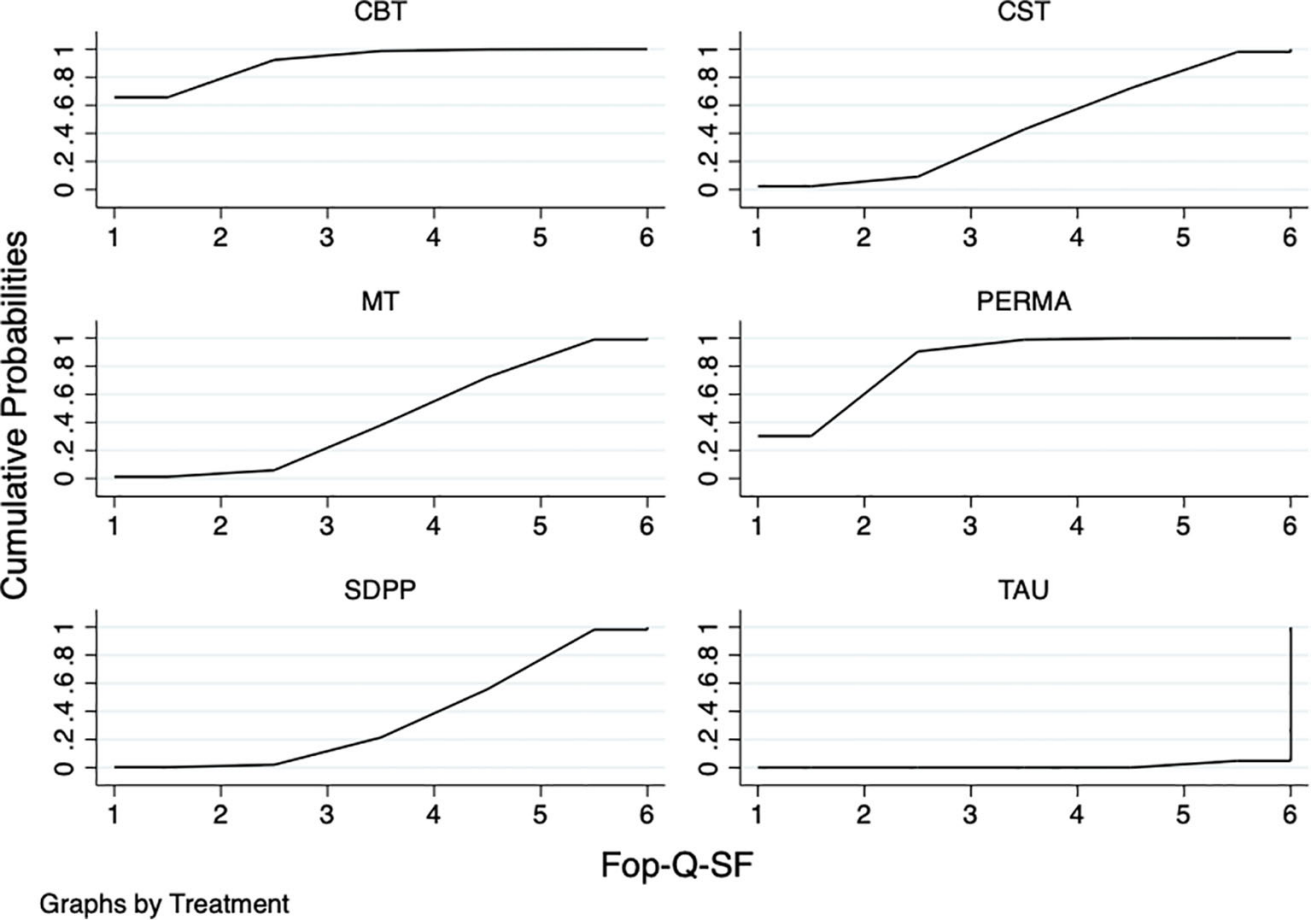
SUCRA plot of different psychotherapies to improve the progression of fear of cancer recurrence. The SUCRA score represents only a probability ranking and does not indicate clinical superiority.

The results of the network meta-analysis showed that: compared with TAU, PERMA(MD=-5.16,95%CI:-6.60~-3.71),CST(MD=-2.88,95%CI:-5.60~-0.16), MT(MD=-2.76,95%CI:-5.18~-0.34), SDPP(MD=-2.30,95%CI:-4.54~-0.06), and CBT (MD=-5.76,95%CI:-7.98~-3.55) were all superior to TAU, and the differences were statistically significant (P<0.05). The results of the SUCRA probability ranking showed that CBT(91.3%) > PERMA(83.9%) > CST(45.0%) > MT(43.3%) > SDPP (35.5%) > TAU(1.0%) (**Table 4**; **Figure 6**).

**Table 4:** NMA of different psychotherapies on different psychotherapies on fear of cancer recurrence progress.

Intervention	PERMA	CST	MT	SDPP	CBT	TAU
PERMA	—	-2.28 (-5.35,0.80)	-2.40 (-5.22,0.42)	-2.86 (-5.52,-0.19)	0.60 (-2.04,3.25)	-5.16 (-6.60,-3.71)
CST	—	—	-0.12 (-3.76,3.52)	-0.58 (-4.10,2.94)	2.88 (-0.62,6.38)	-2.88 (-5.60,-0.16)
MT	—	—	—	-0.46 (-3.76,2.84)	3.00 (-0.28,6.28)	-2.76 (-5.18,-0.34)
SDPP	—	—	—	—	3.46 (0.31,6.61)	-2.30 (-4.54,-0.06)
CBT	—	—	—	—	—	-5.76 (-7.98,-3.55)

PERMA, PERMA Positive Psychotherapy; CST, Couples’s skills training; MT, Mindfulness therapy; SDPP, Self-disclosure in Positive Psychology; CBT, Cognitive-behavioral therapy; TAU, Treatment as usual.

#### Positive psychological capital questionnaire

**Figure 7:**
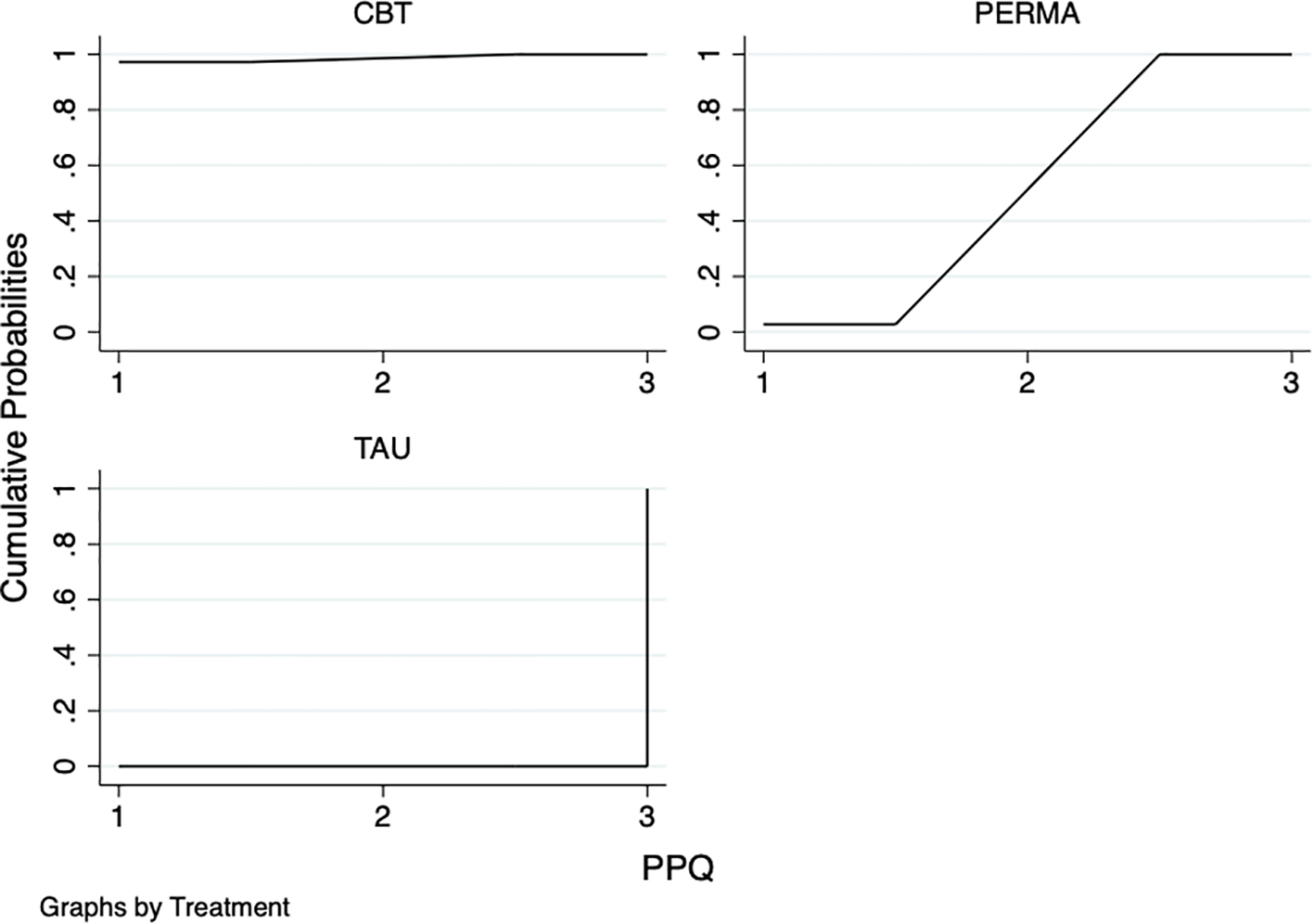
SUCRA plot of different psychotherapies to improve the positive psychological capital questionnaire. The SUCRA score represents only a probability ranking and does not indicate clinical superiority.

The network meta-analysis results showed that compared with TAU, PERMA (MD=-28.20, 95%CI:-33.46~-22.94) was superior, with a statistically significant difference (P<0.05). No statistically significant differences were found between the remaining psychotherapies and TAU (P>0.05). The results of the SUCRA probability ranking indicated that CBT(98.6%) > PERMA(51.4%) > TAU(0.0%) (**Table 5**; **Figure 7**).

**Table 5:** NMA of different psychotherapies on positive psychological capital questionnaire.

Intervention	CBT	PERMA	TAU
CBT	—	21.14 (16.09, 26.19)	-7.06 (-14.35, 0.23)
PERMA	—	—	-28.20 (-33.46, -22.94)

CBT, Cognitive-behavioral therapy; PERMA, PERMA Positive Psychotherapy; TAU, Treatment as usual.

#### Hospital anxiety and depression scale

**Figure 8:**
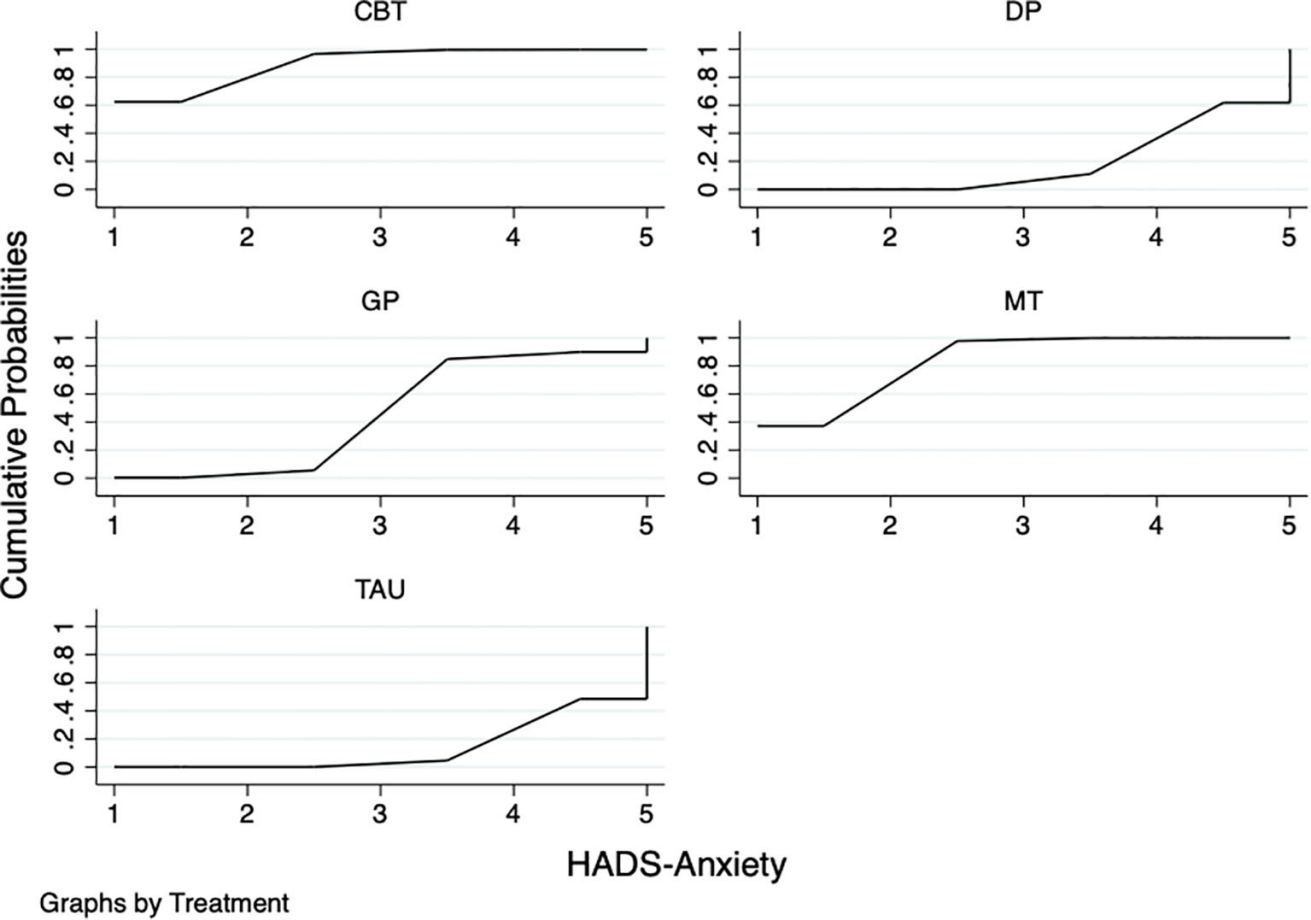
SUCRA plot of different psychotherapies to improve the HADS-anxiety. The SUCRA score represents only a probability ranking and does not indicate clinical superiority.

The results of the network meta-analysis showed that compared with TAU, MT(MD=-2.81,95%CI:-4.34~-1.28) and CBT(MD=-3.25,95%CI: -5.50~-1.00) could relieve anxiety. The differences were statistically significant (P<0.05). In the comparison between GP, DP, and TAU, there were no statistically significant differences (P > 0.05). The results of the SUCRA probability ranking showed that CBT(89.6%) > MT (83.7%) > GP(45.2%) >DP(18.2%) > TAU(13.3%) (**Table 6**; **Figure 8**).

**Table 6:** NMA of different psychotherapies on hospital anxiety and depression scale.

Intervention	GP	DP	MT	CBT	TAU
GP	—	Anxiety: -0.76 (-2.13, 0.61) Depression: -0.41 (-1.82, 1.00)	Anxiety: 2.01 (-0.01, 4.03) Depression: 2.85 (0.71, 4.99)	Anxiety: 2.45 (-0.15, 5.05) Depression: 1.85 (-0.11, 3.81)	Anxiety: -0.80 (-2.12, 0.52) Depression: -0.90 (-2.25, 0.45)
DP	—	—	Anxiety: 2.77 (1.19, 4.35) Depression: 3.26 (1.56, 4.96)	Anxiety: 3.21 (0.93, 5.49) Depression: 2.26 (0.79, 3.73)	Anxiety: -0.04 (-0.41, 0.33) Depression: -0.49 (-0.88, -0.10)
MT	—	Anxiety: -2.77 (1.19, 4.35) Depression: 3.26 (1.56, 4.96)	—	Anxiety: 0.44 (-2.28, 3.16) Depression: -1.00 (-3.18, 1.18)	Anxiety: -2.81 (-4.34, -1.28) Depression: -3.75 (-5.41, -2.09)
CBT	—	Anxiety: 3.21 (0.93, 5.49) Depression: 2.26 (0.79, 3.73)	Anxiety: 0.44 (-2.28, 3.16) Depression: -1.00 (-3.18, 1.18)	—	Anxiety: -3.25 (-5.50, -1.00) Depression: -2.75 (-4.17, -1.33)

GP, Group Psychotherapy; DP, Digital Psychotherapy; MT, Mindfulness therapy; CBT, Cognitive-behavioral therapy; TAU, Treatment as usual.

**Figure 9:**
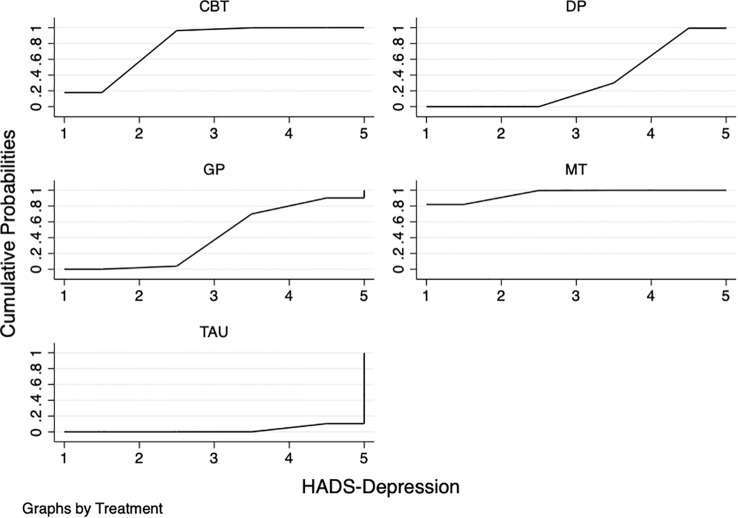
SUCRA plot of different psychotherapies to improve the HADS-depression. The SUCRA score represents only a probability ranking and does not indicate clinical superiority.

DP(MD =-0.49, 95%CI:-0.88~-0.10), MT(MD=-3.75,95%CI:-5.41~-2.09), and CBT(MD=-2.75,95%CI:-4.17~-1.33) could relieve depressive symptoms compared with TAU, and the differences were statistically significant (P<0.05). In the comparison between GP and TAU, there was no statistically significant difference (P>0.05). The results of the SUCRA probability ranking showed that MT(95.1%) > CBT(78.9%) > GP(41.7%) > DP(31.9%) >TAU(2.5%) (**Table 6**; **Figure 9**).

### Subgroup analysis

To evaluate the transitivity assumption of this study, subgroup analyses were conducted based on treatment status within patient characteristics, measurement time points within intervention characteristics, and measurement tools for outcome measures. Since all participants in this study were breast cancer patients at stages 0-III, no subgroup analysis by cancer stage was performed. The results are as follows:

#### Subgroup by measurement time points

**Figure 10:**
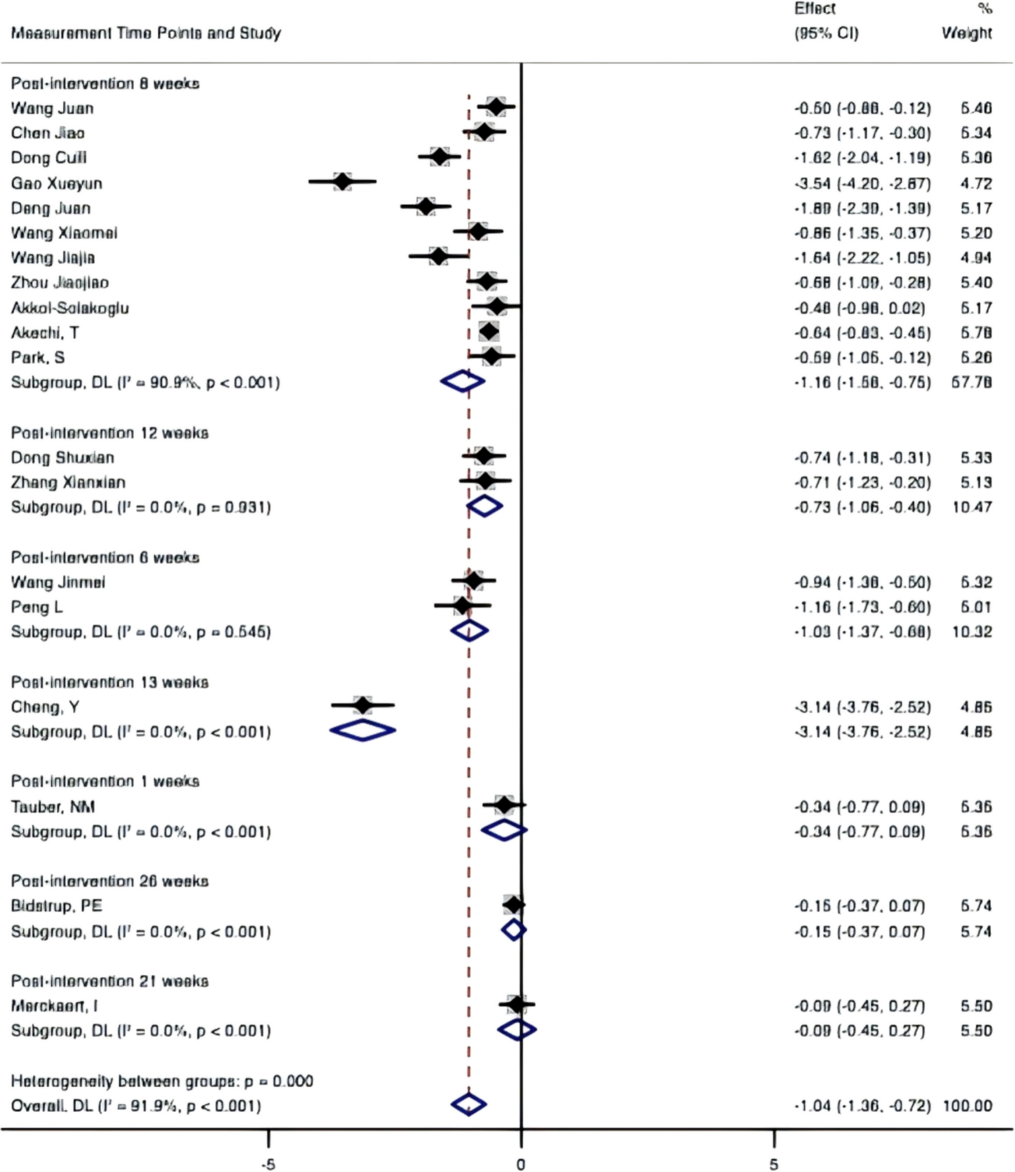
Subgroup by measurement time points.

Stratification by post-intervention follow-up time points (1 week, 6 weeks, 8 weeks, 12 weeks, 13 weeks, 21 weeks, 26 weeks) revealed significant between-group heterogeneity (P = 0.000). The 8-week subgroup exhibited the highest within-group heterogeneity (I²=90.9%,P<0.001), while other subgroups (6 weeks, 13 weeks, 1 week, 26 weeks, 21 weeks) showed no within-group heterogeneity (I²=0%) (**Figure 10**).

Subgroup by treatment status.

**Figure 11:**
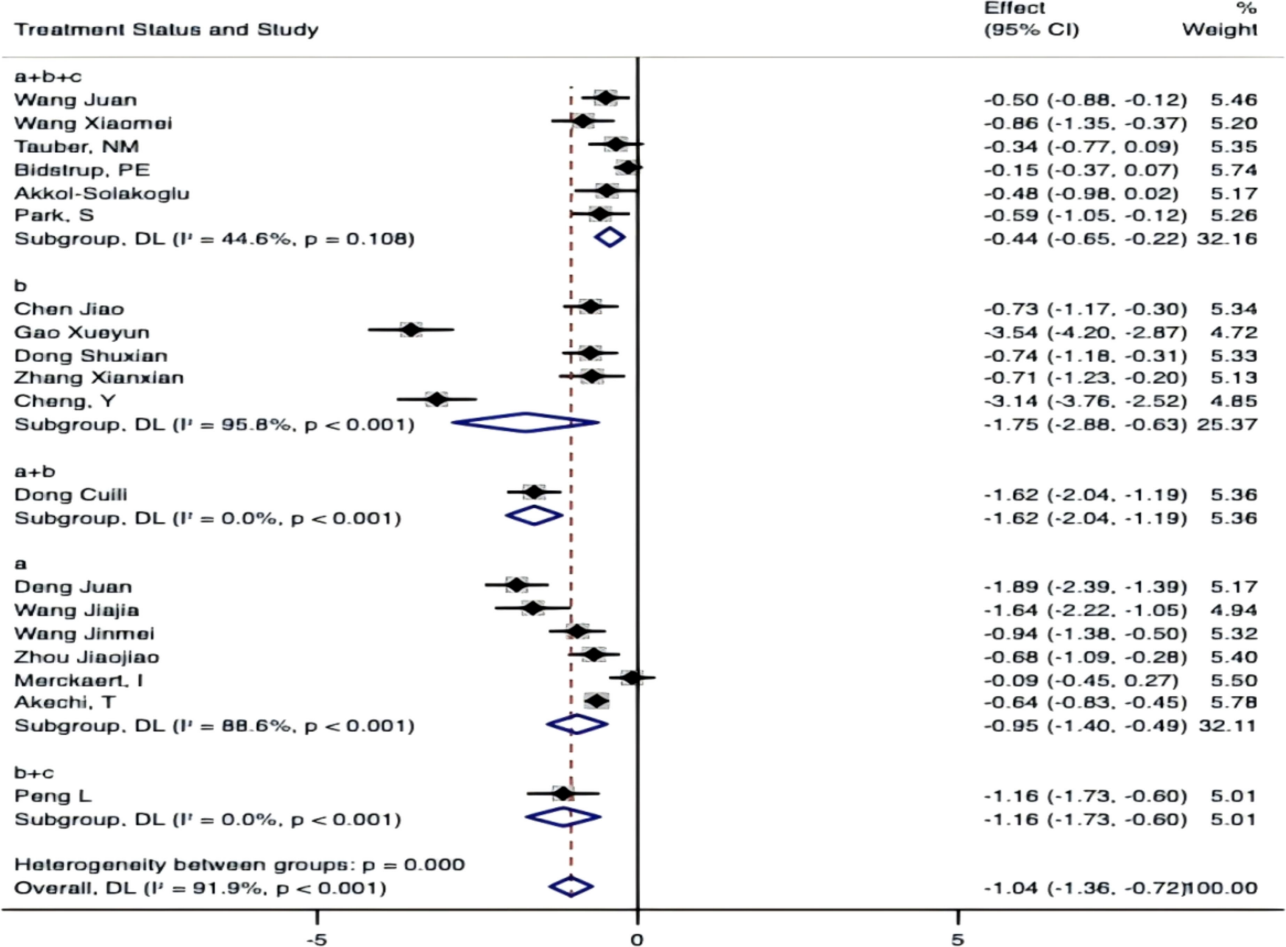
Subgroup by treatment status. a+b+c, Surgical treatment + Chemotherapy + Radiation therapy; b, Chemotherapy; a+b, Surgical treatment + Chemotherapy; a, Surgical treatment; b+c, Chemotherapy + Radiation therapy.

When categorized by treatment status (a:surgical treatment;b:chemotherapy;c:radiation therapy), significant between-group heterogeneity was also observed (P = 0.000). The surgical treatment+chemotherapy+radiation therapy subgroup showed moderate within-group heterogeneity (I²=44.6%, P = 0.108), whereas the chemotherapy subgroup displayed extreme within-group heterogeneity (I²=95.8%, P<0.001), and the surgical treatment+chemotherapy, surgical treatment, and chemotherapy+radiation therapy subgroups showed no within-group heterogeneity (I²=0%) (**Figure 11**).

Subgroup by measurement tools.

**Figure 12:**
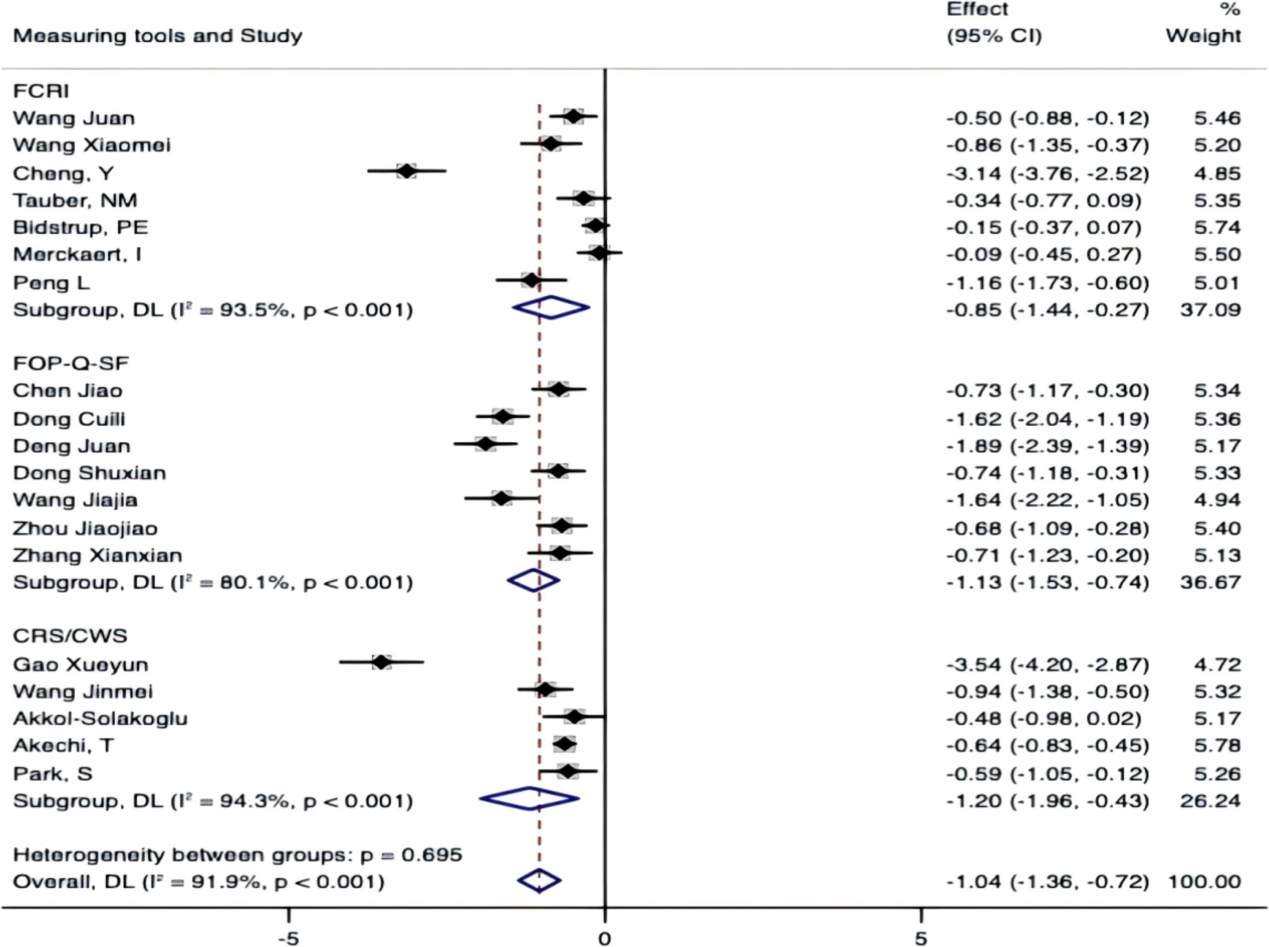
Subgroup by measurement tools.

Subgrouping by measurement tools (FCRI, FOP-Q-SF, CRS/CWS) yielded no significant between-group heterogeneity (P = 0.695), suggesting that measurement tools were not a primary contributor to overall heterogeneity. However, each tool subgroup displayed high within-group heterogeneity (FCRI: I²=93.5%, P<0.001; FOP-Q-SF: I²=80.1%, P<0.001; CRS/CWS: I²=94.3%, P<0.001) (**Figure 12**).

### Sensitivity

**Figure 13:**
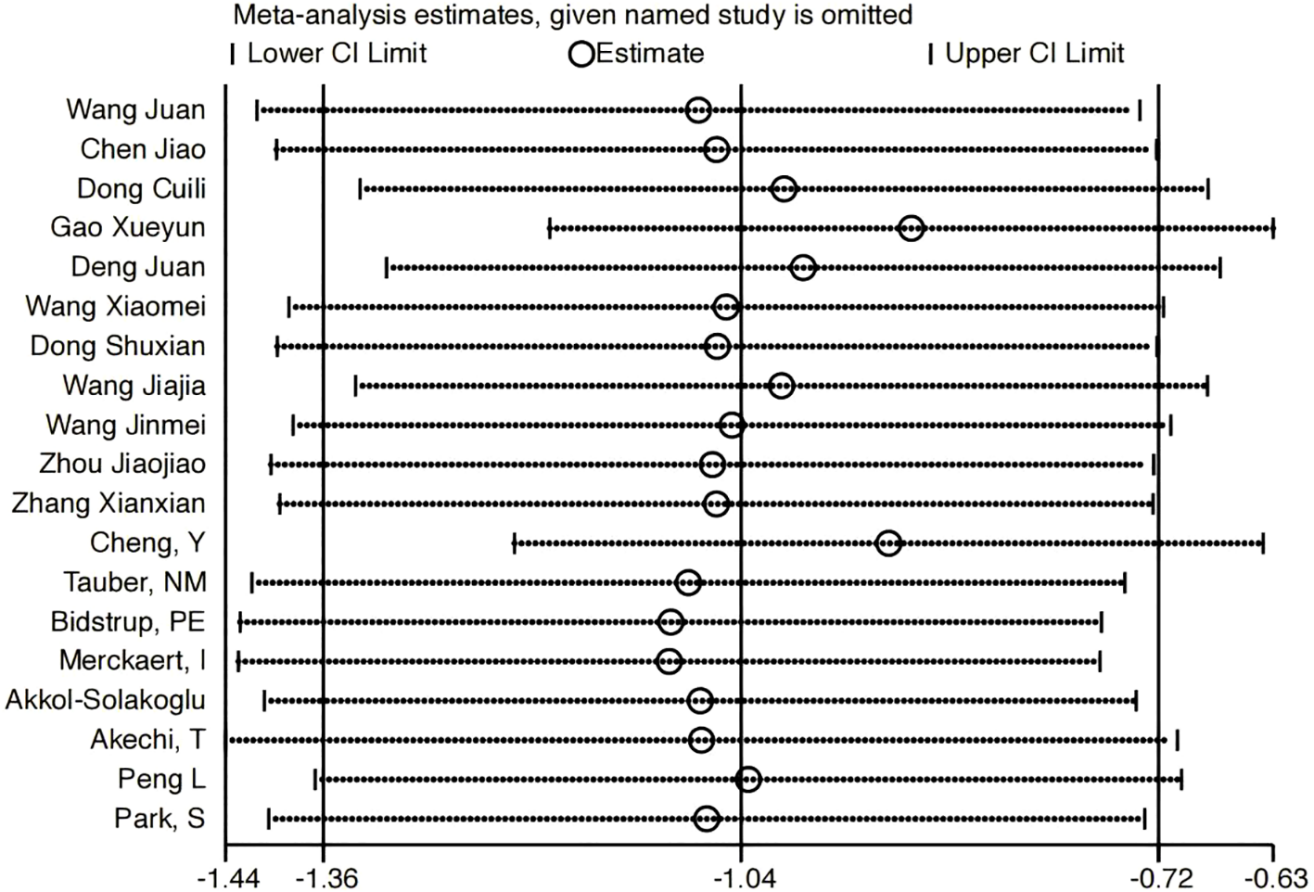
Sensitivity.

To rigorously assess the robustness of our meta-analytic findings, we conducted leave-one-out sensitivity analyses. These analyses confirmed that our core conclusions remained robust, with no single study unduly influencing the pooled effect estimate. When contextualized with our subgroup analysis results, this stability underscores the reliability of our core findings, even in the presence of substantial overall heterogeneity (I²=91.9%, P<0.001) (**Figure 13**).

### Publication bias detection

**Figure 14:**
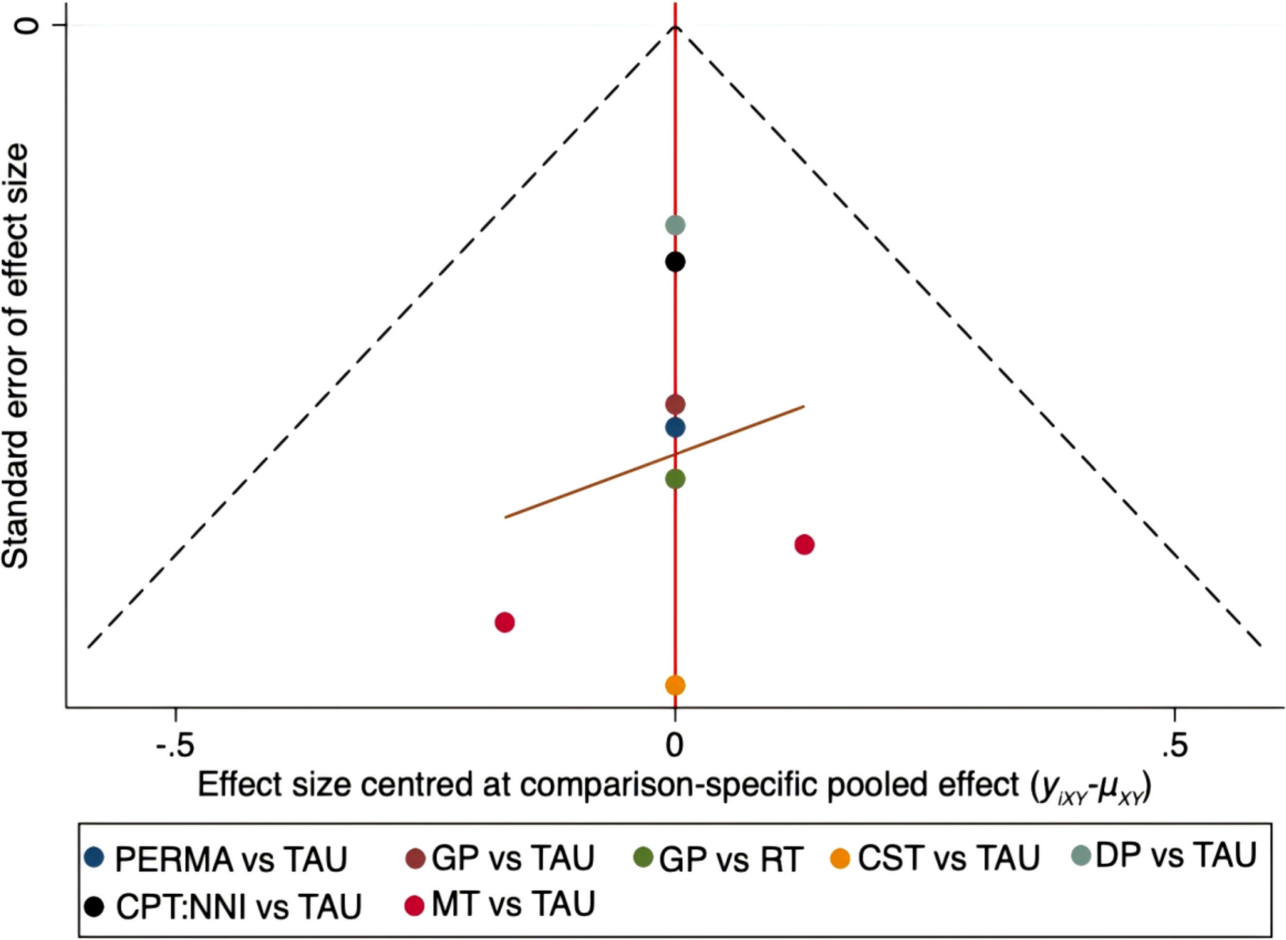
Funnel plot on publication bias of fear of cancer recurrence.

**Figure 15:**
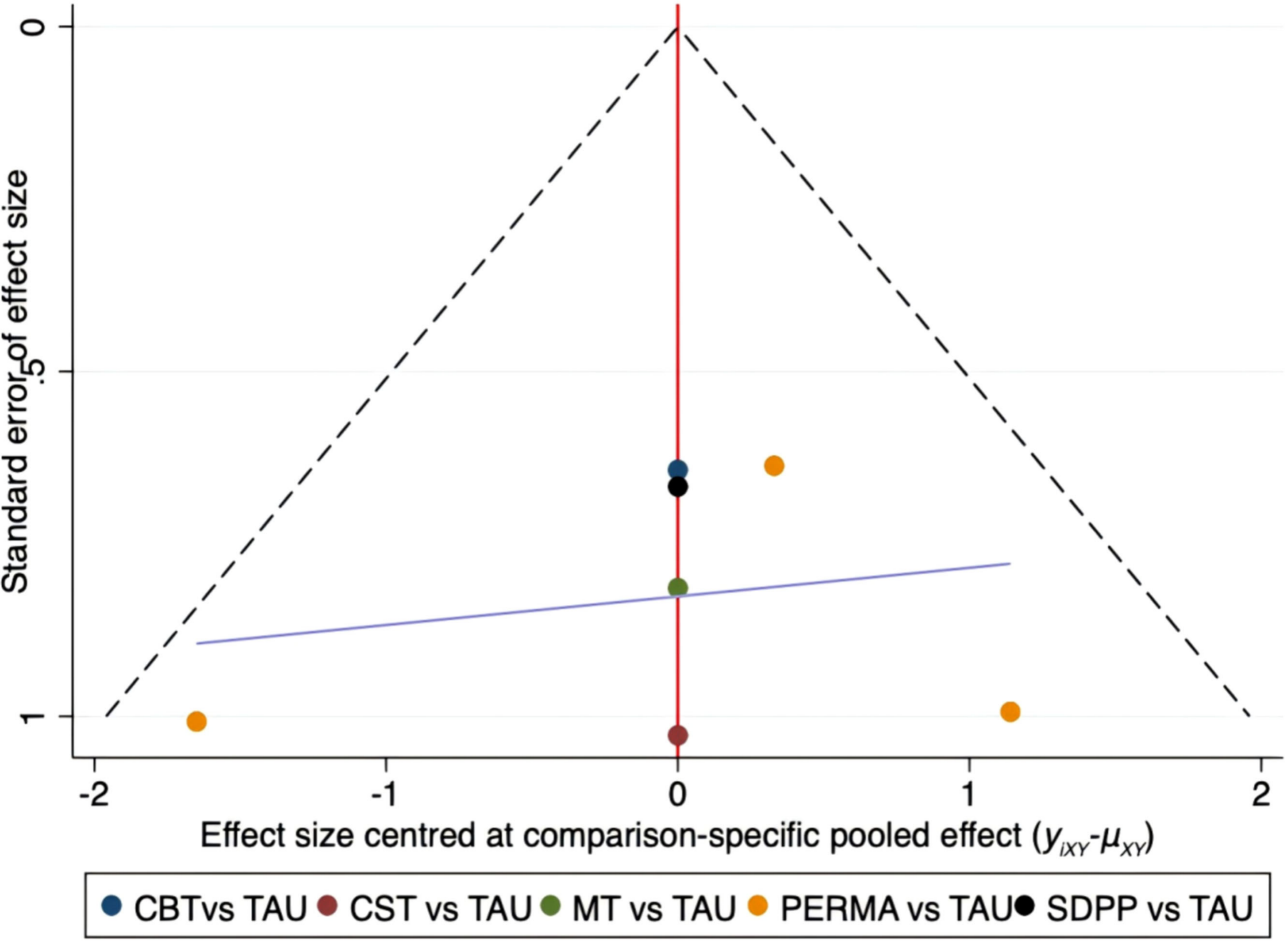
Funnel plot on publication bias of fear of progression questionnaire-short form.

Publication bias analysis was conducted on FCRI and Fop-Q-SF, and funnel plots were drawn. The funnel plots indicated that the scatter points tended to be completely symmetrical, suggesting a low probability of publication bias. The details are shown in **Figures 14**, **15**.

## Discussion

In recent years, the issues of fear of cancer recurrence (FCR) and psychological resilience among breast cancer patients have attracted significant attention. The complexity of the disease itself and its high recurrence rate have instilled anxiety in patients. Many patients are aware that breast cancer can recur several years or even decades after treatment. This uncertainty regarding recurrence has severely affected the physical and mental well-being of patients. The treatment process, psychosocial factors, and economic factors are all risk factors contributing to FCR (26). A study (27) has shown that the treatment process is positively correlated with FCR. The more severe the treatment side - effects, the stronger the patients’ fear of recurrence. Additionally, some literature (28) has pointed out that after patients return to their families and society, the pressure from excessive concern about their condition from relatives and friends, or witnessing the tragic experiences of other patients with recurrence, can exacerbate their fear of recurrence.

FCR has a profound impact on the physical and mental health of patients, including sleep quality, social activities, and mental well-being. It leads to frequent psychological problems such as anxiety and depression, thereby reducing the overall quality of life of patients (29). A study (30) has indicated that breast cancer patients who have been in a long-term state of worrying about cancer recurrence have a significantly higher incidence of psychological disorders such as anxiety and depression. Patients with high psychological resilience are more adept at adopting positive coping strategies to face the various challenges posed by the disease, such as seeking social support and maintaining an optimistic attitude. These strategies not only help alleviate the psychological stress caused by FCR but also enhance patients’ confidence in and compliance with treatment (31, 32). Therefore, paying attention to FCR and psychological resilience is of great significance for the long-term treatment and recovery of breast cancer patients.

The network meta-analysis showed that cognitive-behavioral therapy (CBT) had a probabilistic advantage in alleviating fear of cancer recurrence (Fop-Q-SF), positive psychological capital scores (PPQ), and patients’ anxiety (HADS - Anxiety). This suggests that CBT can be the preferred treatment method for improving FCR and psychological resilience. CBT can assist patients in identifying and correcting irrational cognitions about cancer recurrence. For example, many breast cancer patients have cognitive biases of exaggerating the possibility and consequences of recurrence, believing that recurrence is equivalent to death (33). Through CBT, patients can be guided to analyze the irrationality of their fear-related thoughts and re-evaluate the recurrence risk (34). Meanwhile, it helps patients learn to actively cope with the disease, reduce avoidance behaviors, and thereby enhance their ability to cope with FCR and improve psychological resilience (35).

This study demonstrates that couples’ skills training (CST) has a probabilistic advantage in alleviating FCR among breast cancer patients, with a statistically significant difference compared with conventional psychological therapy (P < 0.05). Therefore, CST is suggested as the optimal psychological treatment for improving FCR. From the perspective of emotional support, deep self-disclosure between spouses allows patients to fully express inner emotions such as fear and worry about cancer recurrence. When spouses patiently listen and respond with understanding, patients can feel accepted and cared for. This strong emotional support can significantly alleviate the loneliness and fear caused by the uncertainty of cancer recurrence (36). A study (37) has shown that positive emotional interactions between spouses, such as daily concern and joint participation in activities, can divert patients’ attention from the fear of recurrence, helping them face the disease with a more positive and optimistic attitude. Through such emotional interactions, patients can obtain psychological comfort, enhance their confidence in coping with the disease, and thus reduce the level of FCR (38–40), which is consistent with the results of this study.

Regarding the concern about cancer recurrence, the results of this study showed that narrative therapy, digital psychological therapy, and mindfulness therapy were all statistically significant compared with conventional psychological therapy (P < 0.05). Among them, narrative therapy had more advantages in improving the concern about cancer recurrence among breast cancer patients. Therefore, narrative therapy is recommended as the optimal psychological treatment method. The core of narrative therapy is to guide patients to use their disease-related experiences as story materials, and reorganize and interpret them to discover new meanings and values, thereby achieving psychological reconstruction (41). During the disease, breast cancer patients experience conflicts in self-image and identity. Narrative therapy guides patients to explore the positive elements of their disease experiences, such as the strength and courage they demonstrated during the treatment process, thus reshaping a positive self-perception (42). In addition, narrative therapy can also assist in the rehabilitation planning of breast cancer patients. When patients actively participate in the rehabilitation planning and witness their progress, they will have more confidence in the recovery of the disease. This positive emotion can greatly relieve the concern about cancer recurrence and effectively improve the concern about cancer recurrence among breast cancer patients (43).

This study showed that CBT and mindfulness therapy were the best psychological treatment methods for improving patients’ anxiety and depression, while digital psychological therapy was meaningful for improving the depression of breast cancer patients but not the optimal measure. Mindfulness therapy includes methods such as mindfulness meditation and mindfulness breathing, which emphasize consciously perceiving the present moment and non - judgmentally accepting one’s feelings and experiences to improve the psychological state of patients. This therapy can help patients better perceive their emotions and strengthen the regulation of negative emotions (44). A study (45) has shown that the use of mindfulness therapy can enhance the psychological resilience of breast cancer patients, thereby alleviating their anxiety and depression, which is consistent with the results of this study.

## Limitations

This study only included Chinese and English literature. The number of studies included in some parts was insufficient, and blinding was not implemented in some of the included studies. As a result, certain biases may exist in the findings.

Moreover, the literature search was limited to specific databases, which might have led to the omission of relevant studies from other sources. Additionally, the included studies varied in terms of sample sizes, patient populations, and study designs. These heterogeneities could potentially influence the generalizability of the results. Future research should consider expanding the scope of literature retrieval, including studies from multiple languages and various publication platforms. Furthermore, the limited number of included studies restricted the depth of subgroup analyses and the exploration of potential effect modifiers.

## Conclusions

Fear of cancer recurrence (FCR) and psychological resilience in breast cancer patients are critical clinical concerns warranting rigorous investigation. Current psychological interventions for this cohort fall into three core categories: (1) psychotherapy-oriented strategies (e.g., cognitive behavioral therapy, group psychotherapy); (2) mind-body regulation techniques (e.g., mindfulness meditation, relaxation training); and (3) comprehensive support-based interventions (e.g., family support programs, informational support). Here, we integrated cognitive-support-relaxation multimodal psychosocial interventions to identify optimal approaches for mitigating FCR and enhancing psychological resilience in breast cancer patients.

Our findings identify cognitive behavioral therapy, narrative therapy, and couples’ skills training as more effective interventions for reducing FCR, while cognitive behavioral therapy and mindfulness therapy emerge as the preferred strategies for enhancing psychological resilience. Notably, digital psychological therapies, PERMA positive psychology interventions, relaxation training, group psychotherapy, and nurse navigation-led comprehensive psychosocial interventions were not top-tier approaches in this analysis but hold considerable potential for further evaluation in large-scale, well-designed prospective studies. Furthermore, subgroup analyses revealed that the efficacy of distinct psychological interventions for FCR in breast cancer patients is modulated by treatment status and post-intervention measurement time points, and these two factors thus require rigorous consideration when selecting and implementing such intervention protocols in clinical practice.

## Data Availability

The original contributions presented in the study are included in the article/supplementary material. Further inquiries can be directed to the corresponding author.
